# Supplementary material for: Purkinje cell misfiring generates high-amplitude action tremors that are corrected by cerebellar deep brain stimulation
Source: eLife. 2020 Mar 17;9:e51928. doi: 10.7554/eLife.51928 (PMC7077982; doi:10.7554/eLife.51928)
Supplement: Figure 1—figure supplement 1—source data 1. [file elife-51928-fig1-figsupp1-data1.docx]

**Figure 1—figure supplement 3—source data 2:** Precision measures, exact p-values, and replicate data relevant to Figure 1—figure supplement 3.

| Figure | Comparator 1 | Comparator 2 | Mean 1 | Mean 2 | SE of diff. | n 1 | n 2 | Summary | Adjusted  P Value |
| --- | --- | --- | --- | --- | --- | --- | --- | --- | --- |
| Figure 1—figure supplement 3a | *Slc32a1^flox/flox^*  Fastigial | *Pcp2^Cre^;Slc32a1^flox/flox^*  Fastigial | 3.656e-4 puncta / µm^2^ | 2.849e-4 puncta / µm^2^ | 1.616e-5 | 16 | 14 | *** | 0.0007 |
|  | *Slc32a1^flox/flox^*  Interposed | *Pcp2^Cre^;Slc32a1^flox/flox^*  Interposed | 4.015e-4 puncta / µm^2^ | 1.982e-4 puncta / µm^2^ | 4.272e-5 | 16 | 14 | ** | 0.0011 |
|  | *Slc32a1^flox/flox^*  Dentate | *Pcp2^Cre^;Slc32a1^flox/flox^*  Dentate | 1.532e-4 puncta / µm^2^ | 7.209e-5 puncta / µm^2^ | 2.865e-5 | 16 | 14 | * | 0.0418 |
| Figure 1—figure supplement 3b | *Slc32a1^flox/flox^*  Fastigial | *Pcp2^Cre^;Slc32a1^flox/flox^*  Fastigial | 2.390% | 1.549% | 0.1074 | 16 | 14 | **** | <0.0001 |
|  | *Slc32a1^flox/flox^*  Interposed | *Pcp2^Cre^;Slc32a1^flox/flox^*  Interposed | 1.959% | 1.042% | 0.2642 | 16 | 14 | * | 0.0123 |
|  | *Slc32a1^flox/flox^*  Dentate | *Pcp2^Cre^;Slc32a1^flox/flox^*  Dentate | 0.7028% | 0.3080% | 0.1361 | 16 | 14 | * | 0.0368 |
| Figure | Comparator 1 | Comparator 2 | Mean 1 | Mean 2 | SEM | n 1 | n 2 | Summary | P Value |
| Figure 1—figure supplement 3c | *Slc32a1^flox/flox^* | *Pcp2^Cre^;Slc32a1^flox/flox^* | 1.350e-3 puncta / µm^2^ | 1.324e-3 puncta / µm^2^ | 1.159e-4 | 9 | 6 | ns | 0.8202 |
| Figure 1—figure supplement 3d | *Slc32a1^flox/flox^* | *Pcp2^Cre^;Slc32a1^flox/flox^* | 8.284% | 9.119% | 0.8126 | 9 | 6 | ns | 0.3229 |
